# First Genome-Scale Metabolic Model of *Dolosigranulum pigrum* Confirms Multiple Auxotrophies

**DOI:** 10.3390/metabo11040232

**Published:** 2021-04-09

**Authors:** Alina Renz, Lina Widerspick, Andreas Dräger

**Affiliations:** 1 Computational Systems Biology of Infections and Antimicrobial-Resistant Pathogens, Institute for Bioinformatics and Medical Informatics (IBMI), University of Tübingen, 72076 Tübingen, Germany; renz@informatik.uni-tuebingen.de (A.R.); lina.widerspick@student.uni-tuebingen.de (L.W.); 2 Department of Computer Science, University of Tübingen, 72076 Tübingen, Germany; 3 Cluster of Excellence ‘Controlling Microbes to Fight Infections’, University of Tübingen, 72076 Tübingen, Germany; 4 German Center for Infection Research (DZIF), Partner site Tübingen, 72076 Tübingen, Germany

**Keywords:** *Dolosigranulum pigrum*, genome-scale metabolic model, *Staphylococcus aureus*, interaction, auxotrophy, nose microbiome

## Abstract

*Dolosigranulum pigrum* is a quite recently discovered Gram-positive coccus. It has gained increasing attention due to its negative correlation with *Staphylococcus aureus*, which is one of the most successful modern pathogens causing severe infections with tremendous morbidity and mortality due to its multiple resistances. As the possible mechanisms behind its inhibition of *S. aureus* remain unclear, a genome-scale metabolic model (GEM) is of enormous interest and high importance to better study its role in this fight. This article presents the first GEM of *D. pigrum*, which was curated using automated reconstruction tools and extensive manual curation steps to yield a high-quality GEM. It was evaluated and validated using all currently available experimental data of *D. pigrum*. With this model, already predicted auxotrophies and biosynthetic pathways could be verified. The model was used to define a minimal medium for further laboratory experiments and to predict various carbon sources’ growth capacities. This model will pave the way to better understand *D. pigrum*’s role in the fight against *S. aureus*.

## 1. Introduction

*Dolosigranulum pigrum* is a rare and rather newly identified opportunistic pathogen [1]. While other microbes, such as *Escherichia coli*, were already detected in the late 19th century [2], *D. pigrum* was first described in 1993 by Aguirre et al. [3]. *D. pigrum* is a Gram-positive, catalase-negative coccus growing in pairs, tetrads, and clusters [3]. In sporadic cases, *D. pigrum* is associated with diseases [1,3,4,5,6,7].

In 2000, the antimicrobial susceptibility and the sources of 27 clinical isolates of *D. pigrum* were determined [8]. The isolation sources ranged from blood and eye cultures from nasopharyngeal swab, sputum, sinus, gastric, and urine specimens to a spinal cord autopsy. The 27 clinical isolates were tested for their susceptibility to 15 antimicrobial agents. *D. pigrum* is a potential pathogen for humans with exceptional resistance to erythromycin but susceptibility to a wide range of other antimicrobial agents [8].

The focus shifted from *D. pigrum* as an opportunistic pathogen to its potential probiotic effect in upper respiratory tract infections in the last years. Together with *Corynebacterium pseudodiphteriticum*, *D. pigrum* was identified as the nasopharyngeal species associated with a healthy upper respiratory tract (URT) and resistance to recurrent ear infections [9]. Multiple studies strengthen this positive association between a healthy URT and *D. pigrum*, especially in children [10,11,12,13,14,15,16,17,18,19]. Several studies indicate a decrease in the abundance of *D. pigrum* after antibiotic treatment [14,18,20], while otopathogenic genera were not affected by antibiotic treatment [21]. Together with the antimicrobial susceptibility study by LaClaire and Facklam [8] and Lopes et al. [22], this might indicate a high sensitivity of *D. pigrum* to antibiotic agents.

*D. pigrum* is relevant for the URT and further parts of the respiratory tract. The abundance of *D. pigrum* is decreased in children with cystic fibrosis (CF) compared to healthy children [20]. *D. pigrum* seems to produce significantly less biomass than the conventional CF pathogen *P. aeruginosa* but is crucial for increasing tolerance of the mixed biofilm to most antibiotics [22,23]. However, the role of *D. pigrum* within the microbial communities in patients with CF is currently still not fully understood [24].

As the human nose is part of the upper respiratory tract, *D. pigrum* also plays a pivotal role in the human nasal microbiota [25]. Additionally to the negative association of *D. pigrum* with *Streptococcus pneumoniae*, it is also negatively associated with *Staphylococcus aureus*. Approximately one-third of the human population is permanently colonized by *S. aureus* [26]. It can cause severe infections with high morbidity and mortality [27]. Its methicillin-resistant strains are one of the most successful modern pathogens [28]. Liu et al. identified *D. pigrum* as a predictor of the presence or absence of *S. aureus* [29]. Brugger et al. strengthened the relevance of *D. pigrum* as a potential probiotic due to its inhibiting effects on *S. aureus*. However, the overall mechanisms behind the inhibition remain unclear. Possible mechanisms include nutrient competition or the excretion of primary or secondary metabolites [25].

Such hypotheses could be tested using genome-scale metabolic models (GEMs) of the organisms of interest, e.g., *D. pigrum* and *S. aureus*. Currently, 114 GEMs of *S. aureus* are available [30], but no single GEM of *D. pigrum* exists. Due to its increasing importance in the community with other microbes, such as *S. aureus*, *S. pneumoniae*, or *P. aeruginosa*, the need for a comprehensive and meaningful GEM is of high interest and high significance.

With a community model of *D. pigrum* and other microbes, its interactions and potential probiotic effect could be elucidated. Such interactions are complex and challenging to understand but vital for successful interventions [31]. Especially for the microbial community in the human gut, several studies already investigated the effect of gene knockouts or the absence of a community member [32,33]. The increasing interest and relevance in studying interactions in microbial communities are also highlighted by the increasing number of available tools for modeling bacterial communities, including OptCom, BacArena, or MICOM [32,34,35].

In this work, we introduce the first genome-scale metabolic model of *D. pigrum* strain 83VPs-KB5. This high-quality model comprises multiple annotations and extensive manual curation steps. It was evaluated and validated using all publicly available experimental data to this date. With this model, several auxotrophies were confirmed and additional auxotrophies were identified. To facilitate future laboratory experiments, we developed a chemically defined minimal medium with all the nutritional requirements to cultivate *D. pigrum*. These new findings will pave the way to better understanding *D. pigrum*’s role in the fight against *S. aureus*.

## 2. Results

The model presented in this article is the first publicly available GEM of *D. pigrum* strain 83VPs-KB5. Based on the latest recommended naming conventions of the community standardization of metabolic models [36], this model is called *i*DPM21RW. DPM is the species indicator and simultaneously the organism’s prefix in KEGG [37]. The curators’ names and the year of curation were chosen as iteration identifiers. This GEM of *D. pigrum* comprises 1241 metabolites in 1668 reactions and are encoded by 622 genes. It includes the three compartments cytosol, periplasm, and the extracellular space, which hold 974, 55, and 17 reactions, respectively, excluding transport and exchange reactions. Memote is a metabolic model testing suite that determines for each tested GEM an independent and comparable score within a comprehensive overview. Standardized metabolic model tests and the evaluation of a model’s annotations constitute the score [38]. The final Memote score of *i*DPM21RW amounts to 86%. For comparison, the GEM *i*ML1515 of *Escherichia coli* [39], for which the first version was published in [40], became steadily updated and improved over the last 20 years by the modeling community and has now reached a Memote score of 91%.

### 2.1. Properties of the Constructed GEM

The basis for the manual extension was the draft reconstruction automatically curated with CarveMe [41]. It only requires an annotated genome file of the organism of interest. In a simple command line interface, the model can be “carved”. Other tools for the automated reconstruction of GEMs exist besides CarveMe, such as ModelSEED [42], gapseq [43], or KBase [44]. We chose CarveMe as a curation tool as it accesses the BiGG Models database [45] and uses its identifiers. These identifiers are required for subsequent successful use of the ModelPolisher [46] for adding extensive annotations. ModelSEED and gapseq both use ModelSEED identifiers, and thus, applying the ModelPolisher is currently not feasible.

**Figure 1:**
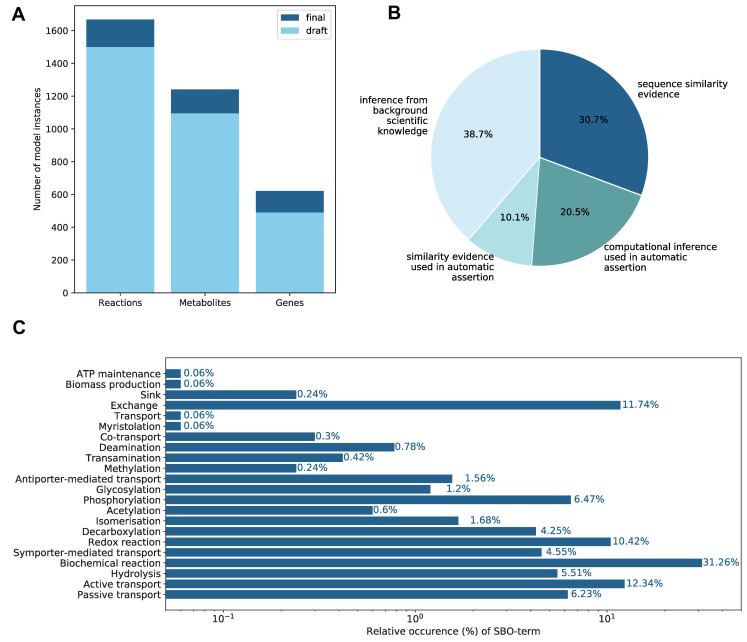
Properties of the genome-scale metabolic model (GEM) *i*DPM21RW. This figure illustrates various model properties. (**A**) The number of model instances in the draft and the refined final reconstruction is indicated. In total, 132 genes, 169 reactions, and 146 metabolites were added to the final reconstruction. (**B**) Evidence and Conclusion Ontology (ECO) terms indicate the confidence of inclusion for the model’s reactions. Increasing color intensity corresponds to increasing confidence. (**C**) Systems Biology Ontology (SBO) terms were used to annotate the models’ reactions further [57]. The axis of the relative occurrence is given as a log scale.

The initial draft reconstruction from CarveMe included only 1499 reactions, 1095 metabolites, and 632 genes. Despite the first impression of a decrease in the number of genes, it needs to be stated that 142 genes were included twice in the initial draft model: once with the prefix G_ and once without this prefix. The duplicated genes were removed, and the 620 genes in the final reconstruction is the number of unique genes. This means that 132 additional genes, 169 reactions, and 146 metabolites were added to the model during the whole manual refinement process. During manual extension based on the KEGG database, 161 reactions, 143 metabolites, and 129 genes were added to the model. An overview of these numbers is given in Figure 1. Metabolic models may contain thermodynamically impossible energy-generating cycles. These models can charge currency metabolites such as adenosine triphosphate (ATP) or reduced nicotinamide adenine dinucleotide phosphate (NADPH) without nutrient consumption [47]. The model *i*DPM21RW was evaluated for the production of 15 energy metabolites while no nutrients were available. None of the tested energy metabolites were produced, and thus, the final model does not contain energy-generating cycles. Of the 1499 reactions, 6.23% are blocked reactions, which means that they cannot carry any flux during flux variability analysis (FVA):x. These blocked reactions might be indicators of knowledge gaps.

#### 2.1.1. Mass and Charge Imbalances

The initial draft model had 858 mass and/or charge imbalanced reactions. After manual refinement of these mass and charge imbalances, more than 82% of the 858 imbalanced reactions were balanced. This increase in balanced reactions is also confirmed by Memote when looking at the mass and charge balance score: The mass balance score increased from 52.7% to 95.6%, and the charge balance score increased from 43.2% to 93.3%. However, 137 reactions were still mass and/or charge imbalanced, none of which were blocked reactions. With novel insights into metabolites’ protonation statuses, the actual participation of metabolites in these reactions and their accurate stoichiometry, and further manual refinement, this number might be reduced even further.

#### 2.1.2. Annotations

The model comprises annotations to various databases. These annotations were added using ModelPolisher [46] and extended manually. For the model reactions, cross-references to the databases MetaNetX [48], Biochemically, Genetically, and Genomically Structured (BiGG) Models [45], UniProt [49], Kyoto Encyclopedia of Genes and Genomes(KEGG) [37], RHEA [50], and BioCyc [51] are included, as are the corresponding EC-numbers, where available. The annotations of the model metabolites contain cross-references to the databases KEGG [37], BiGG [45], BioCyc [51], the Human Metabolome Database (HMDB) [52], MetaNetX [48], and lipidmaps [53]. The gene annotations contain cross-references to KEGG [37] and the NCBI protein database [54].

Additionally, all genes, metabolites, and reactions were further annotated with a term from the Systems Biology Ontology (SBO) [55]. All metabolites were assigned the SBO term SBO:0000247 for “simple chemical”, and all model genes received the SBO term SBO:0000243 coding for “gene”. In total, 22 different SBO terms were assigned to the reactions. The most prominent SBO term with a relative abundance of 31.32% is the SBO:0000176, coding for “biochemical reaction”. All other SBO terms describe more precisely the biochemical reactions, such as the SBO term SBO:0000216 with a relative abundance of 6.5%, coding for a “phosphorylation” reaction. The relative occurrence of all 22 SBO terms is depicted in Figure 1.

The model reactions were further annotated using terms from the Evidence and Conclusion Ontology (ECO) [56]; 38.7% of the model reactions were inferred from background scientific knowledge, 10.1% had similarity evidence, 20.5% held a computational inference, and 30.7% even had sequence similarity evidence. The overall occurrence of the ECO terms is displayed in Figure 1.

#### 2.1.3. Biomass Objective Function

CarveMe creates a general biomass objective function (BOF) during the curation process [41]. This initial BOF was updated using BOFdat [58]. BOFdat is a Python package to generate and improve a BOF based on organism-specific experimental data. In three steps, the stoichiometric coefficients for major macromolecules, inorganic ions and coenzymes, and other species-specific metabolic biomass precursors were calculated and incorporated into the BOF. With the help of the DNA sequence of *D. pigrum*, five stoichiometric coefficients associated with the macromolecule DNA (deoxyadenosine triphosphate (dATP), deoxythymidine triphosphate (dTTP), deoxycytidine triphosphate (dCTP), deoxyguanosine triphosphate (dGTP), and diphosphate)were updated using the first step of the BOFdat algorithm. In the second step of the BOF dat algorithm, the coefficients of inorganic ions and coenzymes were calculated and updated based on macromolecular weight fractions. Fifteen stoichiometric coefficients associated with coenzymes and inorganic ions were updated, and nine were additionally integrated into the BOF. The coefficients of other macromolecules, such as RNA, proteins, or lipids, could not be updated due to a lack of available experimental data. The same was found for the stoichiometric coefficients of other species-specific metabolic biomass precursors as no required gene essentiality data was available. All metabolites included in the BOF, and their stoichiometric coefficients are listed in the supplementary Table S2.

#### 2.1.4. Subsystems and Groups

The group plugin is available from SBML Level 3 [59]. In total, 82 subsystems were added to the plugin as groups. Reactions associated with these subsystems or pathways were added as members to the respective groups. It needs to be highlighted that the subsystems and pathways were extracted from the KEGG database [37]. Thus, only reactions with annotated KEGG identifiers could be mapped to the respective groups. Among the three groups with the most members and, thus, reactions is the subsystem of metabolic pathways with 411 members, the group of biosynthesis of secondary metabolites with 95 members, and the subsystem of microbial metabolism in diverse environments with 79 associated reactions.

### 2.2. Evaluating Auxotrophies and Predicted Biosynthesis

After creating and refining a draft reconstruction and its conversion into a mathematical model, the model needs to be verified, evaluated, and validated. In this step, the model-predicted phenotypes are compared with the experimental data [60]. Brugger et al. predicted the biosynthesis, uptake, and degradation of amino acids, carbohydrates, polyamines, and enzyme cofactors in eleven *D. pigrum* strains by evaluating their genetic content [25]. COBRApy [61] was used for all evaluation steps.

#### 2.2.1. Auxotrophies and Biosynthesis

Brugger et al. identified a methionine auxotrophy in all evaluated *D. pigrum* strains. In our model, growth without methionine supplementation was initially possible, indicating the potential for model adaption and refinement. Nineteen reactions were associated with methionine, which were all carefully checked. We identified and removed four reactions without evidence in KEGG [37], BioCyc [51], or a significant hit in a BLAST search [62]. With these alterations, the model is now incapable of producing methionine, as Brugger et al. observed in their study [25]. An ATP-binding cassette (ABC) transporter for the uptake of methionine is present in the model.

*D. pigrum* has a likely auxotrophy for arginine [25]. We could confirm this observation with our in silico predictions.

Further auxotrophies for the polyamines putrescine and spermidine were predicted [25]. We could also confirm these observations based on our in silico simulations. Additionally, the identified putative ABC-type spermidine transporter and the putative putrescine transporter were already included in the model.

The predicted biotin auxotrophy was initially not observed in the model. For that reason, two biosynthesis reactions were removed from the model, both of which did not have gene–protein reaction (GPR) associations. Instead, the biotin energy-coupling factor (ECF) transporter was added. Brugger et al. identified a biotin-protein ligase in two of the eleven investigated strains. We found the gene for the biotin-protein ligase in the genome of *D. pigrum* strain 83VPs-KB5. Thus, the respective reaction was added to the model.

The last predicted auxotrophy pertained to nicotinic acid (niacin) [25]. This auxotrophy was also observed in the in silico simulations. The identified transporter [25] was already present in the model. The same was found for additional reactions in the conversion of niacin or nicotinamide to NAD^+^ and NADP^+^ with their respective genes. Only one reaction was adapted, as the described enzyme was reclassified into another Enzyme Commission (EC) number with slightly different reactants. The reaction now additionally requires ATP and water instead of a proton and produces adenosine diphosphate (ADP) and a phosphate.

*D. pigrum* is capable of synthesizing l-glutamine from l-glutamate [25]. All required reactions are included in the model, and in silico simulations verify the production of l-glutamine. All predicted auxotrophies and biosynthesis are summarized in Table 1.

**Figure 2:**
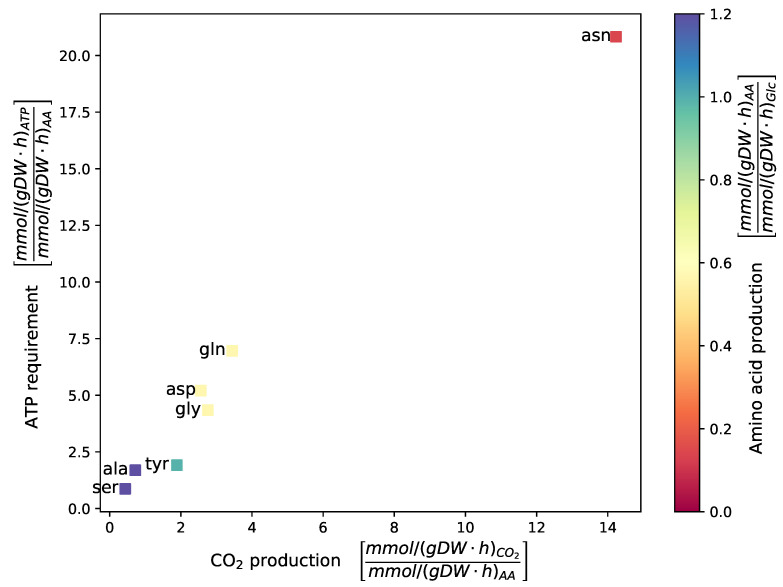
Amino acid production in *D. pigrum*. The exchange reaction of the amino acid of interest was closed to investigate the amino acid production capacity of *D. pigrum* in silico. A sink reaction of the respective amino acid was optimized while maintaining the growth rate at a fixed value of 0.2 mmol/(gDW · h) and maximum growth rate 0.278 mmol/(gDW · h). Only the seven shown amino acids could be synthesized de novo. For every amino acid, the ATP requirement and the CO_2_ production were calculated. The color indicates the amino acid production rate concerning the carbon source (glucose). Amino acids are shown with their respective three-letter code.

Several auxotrophies in *D. pigrum* are already reported in the literature [25]. Thus, we investigated further amino acid auxotrophies or de novo biosynthesis capabilities in silico. As seen in Figure 2, only the seven amino acids l-alanine, l-aspartate, l-glutamine, glycine, l-serine, l-asparagine, and l-tyrosine could be synthesized de novo in our simulation. For all other amino acids, *D. pigrum* seems to be dependent on external sources.

#### 2.2.2. Carbohydrate Metabolism

**Figure 3:**
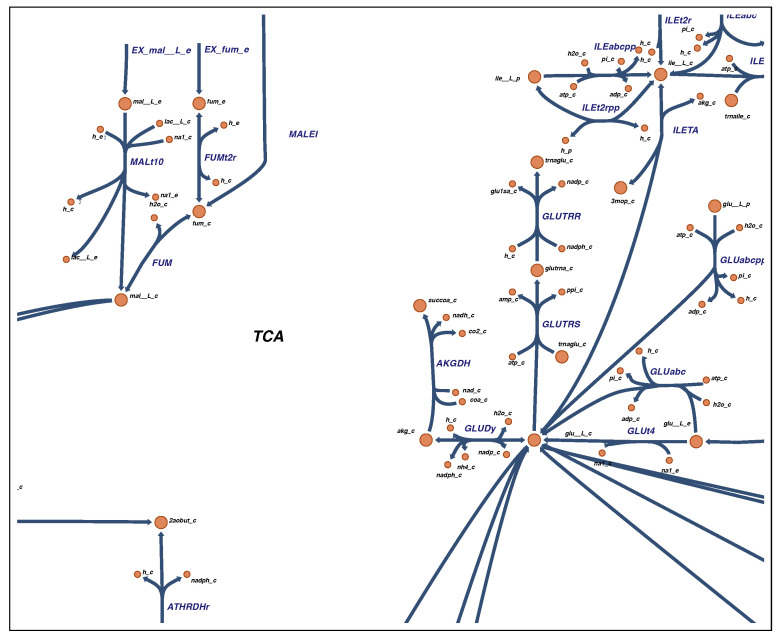
Missing TCA cycle in *D. pigrum*. As predicted by Brugger et al., *D. pigrum* does not have a tricarboxylic acid (TCA) cycle but only two associated reactions. The two reactions are the fumarate-hydratase (FUM) and the TCA-associated dihydrolipoyl dehydrogenase (AKGDH). The map was drawn using Escher [63]. See the Supplementary Materials for a complete map.

Besides auxotrophies, Brugger et al. also investigated the carbohydrate metabolism of eleven *D. pigrum* strains using functional genomic predictions [25]. They found that there is no tricarboxylic acid (TCA) cycle in *D. pigrum*. Our in silico investigations confirmed this finding: only the two reported reactions catalyzed by fumarate-hydratase (FUM) and the TCA-associated dihydrolipoyl dehydrogenase (AKGDH) are present in the model. Those two reactions are illustrated in Figure 3.

Brugger et al. identified V-type ATPases in all investigated strains, which can hydrolyze but not synthesize ATP [25]. The model *i*DPM21RW does not currently include any V-type ATPase as there is no corresponding reaction in the BiGG Models database [45].

The authors investigated anaerobic respiratory reductases and did not identify butyryl-CoA-reductases [25]. *i*DPM21RW does not contain the corresponding reaction BTCOARx, confirming the findings by Brugger et al.

Further investigations concerned ten reactions from glycolysis, including glucokinase, phosphoglycerate kinase, and pyruvate kinase. All ten reactions were confirmed with *i*DPM21RW. Additionally, Brugger et al. predicted the presence and absence of various enzymes relevant for homofermentation to lactate. Each enzyme and its corresponding reaction were checked in our model.

In the last step, Brugger et al. predicted that putative sialidases utilize sialic acids. Sialic acids comprise a family of monosaccharides with a nine-carbon backbone and significant structural diversity [64]. Currently, no sialidase or sialic acidis is present in our model. As more knowledge about *D. pigrum* and its potential utilization of sialic acids becomes available, the corresponding metabolites and reactions can be included in the model.

### 2.3. Evaluating Growth Capabilities

*D. pigrum* was isolated from the sputum, sinuses, the nasopharyngeal tract, blood, and the gastric tract [8]. Thus, it can be assumed that *D. pigrum* can grow in these habitats. The growth of *i*DPM21RW was simulated in chemically defined media, including synthetic nasal medium (SNM) [65], synthetic cystic fibrosis medium (SCFM) [66], an adapted blood medium [67,68], and a gut medium [69,70]. Within these media, the growth rate should not exceed the growth rate of the fastest growing organism, namely *Vibrio natriegens*, with a doubling time of 14.8 min [71], resulting in a flux through the biomass reaction of 2.81 mmol/(gDW · h). Thus, a growth rate below this threshold is considered to be realistic [38].

#### 2.3.1. Growth in SNM

*D. pigrum* is known to grow in the human nose [25]. With the help of the chemically defined synthetic nasal medium (SNM), which mimics the nasal habitat [72], the in silico growth of *D. pigrum* was tested in this niche. However, without additional metabolites, *D. pigrum* did not show any growth in a single culture. We first added the already identified amino acids to the medium, for which *D. pigrum* has auxotrophies: l-isoleucine and l-methionine. Additionally, we identified auxotrophy for meso-2,6-diaminoheptanedioate. This metabolite is required for peptidoglycan metabolism. Despite extensive literature research, including database searches on the KEGG database [37] and BioCyc [51], we could not identify any biosynthetic pathway, suggesting either a knowledge gap or a, so far, unknown auxotrophy. After those three additions to the medium, the model predicted a realistic growth rate of 0.2824 mmol/(gDW · h) in the SNM.

#### 2.3.2. Growth in SCFM

*D. pigrum* is also reported to play a role in CF patients, although its role within the microbial community is currently not fully understood [24]. A chemically defined medium is available, mimicking the lungs of patients with CF. The in silico growth capabilities of *D. pigrum* in this synthetic cystic fibrosis medium (SCFM) were evaluated. Similar to the in silico growth of *D. pigrum* in the SNM, the bacterium did not grow in the SCFM without supplementing additional metabolites. Since we expect the trace elements manganese, zinc, copper, cobalt, and nickel to be contained in the medium even without explicit addition, they are not further examined here. The trace elements were simply added to the medium definition. The other required metabolites were riboflavin, thiamine, nicotinate, 4-aminobenzoate, and, as in the SNM, meso-2,6-diaminoheptanedioate. In their preprint from 2019, Brugger et al. stated that all eleven investigated strains of *D. pigrum* lacked genes for the biosynthesis of thiamine and the de novo synthesis of niacin/nicotinate/nicotinamide [73]. For riboflavin, ten of the eleven strains lacked the synthesis cluster of riboflavin. Our model strain *D. pigrum* 83VPs KB was, however, not among the investigated strains. A literature search in several databases, including KEGG [37] and BioCyc [51], and BLAST searches for relevant biosynthetic enzymes did not reveal any hits for the synthesis of thiamine, riboflavin, and nicotinate, confirming the findings of Brugger et al. For the metabolite 4-aminobenzoate, no information was found in the literature. In KEGG [37] and BioCyc [51], the metabolite was reported in *D. pigrum*, but no synthesis pathways were available. No significant BLAST hits were detected for the enzyme aminodeoxychorismate lyase, which catalyzes the synthesis of 4-aminobenzoate. After adding the required metabolites to the medium, the growth rate of *D. pigrum* in SCFM was 0.2824 mmol/(gDW · h).

#### 2.3.3. Growth in the Blood Medium

*D. pigrum* was isolated from blood samples and even cultivated in aerobic and anaerobic blood culture bottles [1,8]. A chemically defined medium simulating the human blood is available and was used for the in silico simulations [67]. This medium definition was slightly modified and adapted [68]. For the SCFM medium, the trace elements manganese, zinc, copper, cobalt, and nickel are required for growth but are not further investigated here and are only added to the medium definition. Analogously, the compounds 4-aminobenzoate and meso-2,6-diaminoheptanedioate are required for growth, as *D. pigrum* seems to be auxotrophic for those compounds. The in silico simulations predicted a realistic growth rate of 1.908 mmol/(gDW · h) with these metabolites.

As stated above, *D. pigrum* can grow anaerobically in blood cultures. Despite diverse approaches, we could not yet simulate these conditions in our in silico model. There is still much to discover about *D. pigrum*, and with additional information and laboratory experiments, the model could be extended to simulate anaerobic growth in blood cultures.

#### 2.3.4. Growth in the Gastrointestinal Tract

The growth of *D. pigrum* was simulated in the gastrointestinal tract. A defined medium of the European diet from the Virtual Metabolic Human (VMH) database was used for this purpose [69,70]. As in the previously tested media, trace minerals, such as manganese, cobalt, zinc, nickel, and sulfate, were missing in the defined gut medium. The compounds 4-aminobenzoate and meso-2,6-diaminoheptanedioate were again required to enable growth aerobically with a growth rate of 1.088 mmol/(gDW · h).

#### 2.3.5. Definition of a Minimal Medium for *D. pigrum*

The previous analysis of *D. pigrum*’s growth behavior and the investigated auxotrophies indicate specific requirements for its environment and successful colonization. To obtain a detailed picture of all environmental requirements for successful growth, we defined a minimal medium for the growth of *D. pigrum* with the help of *i*DPM21RW (Supplementary Table S3). This minimal medium contains 33 metabolites, which are all listed in Table 2. It includes the 13 amino acids that cannot be synthesized de novo (also see Figure 2) and 13 trace minerals. As a carbon source, D-glucose was chosen. However, in the following section, the growth on different carbon sources is investigated. The three vitamins thiamine (vitamin B_1_), riboflavin (vitamin B_2_), and niacin (vitamin B_3_) are also required to enable growth. 4-aminobenzoate was already mentioned several times to be crucial for *D. pigrum*’s growth. For this reason, it was also included in the minimal medium. The same was found for meso-2,6-diaminoheptanedioate, which was also added to the minimal medium definition. Finally, oxygen is also required for the growth simulations, as anaerobic growth is not yet enabled. Within this minimal medium, the simulated growth rate amounts to 0.2784 mmol/(gDW · h).

#### 2.3.6. Growth on Different Carbon Sources

**Figure 4:**
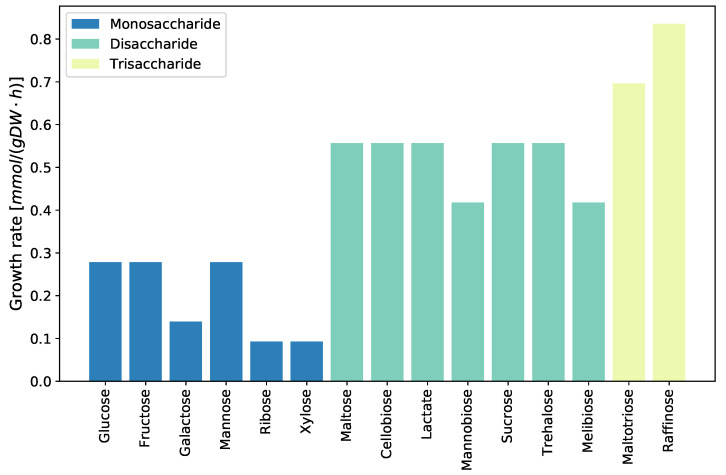
Growth on different carbon sources. *D. pigrum*’s ability to utilize different carbon sources was investigated using the previously defined minimal medium. The available mono-, di-, and trisaccharides were examined concerning the resulting growth rate. As expected, trisaccharides result in a higher growth rate compared to di- and monosaccharides.

Little is known about *D. pigrum*. The previous analysis confirmed several auxotrophies and biosynthetic capacities. To further evaluate the metabolic capabilities, the growth on different carbon sources within the previously defined minimal medium was evaluated. The uptake rate of each tested carbon source was set to 10 mmol/(gDW · h). The available mono-, di-, and trisaccharides were tested as sole carbon sources, as seen in Figure 4. As expected, the growth rate increases with increasing amounts of carbon available. Glucose, fructose, and mannose allow the best growth rates for simulations on monosaccharides as sole carbon sources.

### 2.4. Visualization

A comprehensive map of *D. pigrum*’s metabolism was drawn using Escher [63]. Since a figure would not appropriately capture its large size, the map is included as Supplementary Figure S1 of this publication.

## 3. Discussion

In this work, we generated *i*DPM21RW: the first genome-scale metabolic model of *Dolosigranulum pigrum*. The basis for the manual extension was the draft reconstruction automatically curated with CarveMe [41].

Models curated by ModelSEED and gapseq could be used to extend the already existing model *i*DPM21RW further. This procedure, however, can be challenging because identifier mapping still holds several difficulties. For this purpose, correct and extensive annotations are indispensable. During curation, we put particular focus on the annotations of reactions, metabolites, and genes. Extensive annotations can hold cross-references to other databases, which facilitates the comparability and interoperability of *i*DPM21RW with models from other databases.

ModelPolisher annotates model instances, such as reactions, metabolites, or compartments but not the genes because they are organism- or even strain-specific. Therefore, the manual addition of gene annotations was required. This was a challenging step because the gene annotations should be strain-specific KEGG identifiers. A direct mapping between the NCBI protein identifiers and the KEGG identifiers was not possible since the NCBI protein identifiers often corresponded to so-called “MULTISPECIES” entries that are not uniquely associated with *D. pigrum*. Reaction or metabolite identifiers are often from different databases, and as already mentioned, mapping is challenging. Strain-specific gene identifiers are, however, sometimes more comfortable to map with the corresponding gene and protein annotation files, and the locus-tag information included. This simplifies model comparisons on gene level.

We added cross-references to several other databases and Systems Biology Ontology(SBO) and Evidence and Conclusion Ontology (ECO) terms. ECO terms [56] provide information about the curator’s confidence about a reaction’s inclusion into the model. Confidence scores were previously defined by Thiele and Palsson [60] and the Constraint-Based Reconstruction and Analysis (COBRA) Toolbox. Thiele and Palsson’s confidence score 0 indicates the lowest confidence and 4 indicates the highest confidence with biochemical data evidence. To avoid confusion using only numbers, we decided to use ECO terms. These terms are uniquely defined and can directly be accessed via the Minimal Information Requested in the Annotation of Models (MIRIAM) registry initiative at identifiers.org (accessed on 7 April 2021) [74]. Each reaction was assigned one unique ECO term. However, multiple genes can occur within a GPR. We decided to use a conservative approach and to assign the lowest ECO term of all genes to the reaction. One could also think of assigning the highest identified ECO term, but this might require additional manual verification to avoid inducing false confidence.

The biomass objective function (BOF) was improved using the only available omics data, namely genomics. No transcriptomics, proteomics, or lipidomics data are available, which could be used to further improve the BOF and the model itself by adding detected metabolites, reactions, and genes.

Multiple auxotrophies are reported in *D. pigrum*. Brugger et al. predicted that no tricarboxylic acid ( TCA) cycle is present [25]. The TCA cycle belongs to the most important central metabolic pathways for energy conservation and biosynthesis of key cellular intermediates, including the amino acid biosynthesis [75]. Thus, it seems not surprising that *D. pigrum* has several auxotrophies, especially for amino acids, resulting from the lacking TCA cycle. *D. pigrum* is not the only microbe missing parts of the TCA cycle. A large number of bacteria are reported to have incomplete or unusual TCA cycles [76,77]. This incompleteness or even absence of the TCA cycle might go back to adaptions to the organism’s metabolic lifestyle [76]. However, it might also be the case that apparently “missing” genes are only missing in genome analysis but are revealed in actual biochemical experiments [77]. The observations of Brugger et al. are based on functional genomic prediction, and model curation is based on the genome sequence of *D. pigrum*. Biochemical experiments are required to either confirm the missing TCA cycle or refine the model by adding newly identified reactions.

Further auxotrophies concerned polyamines and vitamins. The polyamines spermidine and putrescine are synthesized from l-arginine and l-methionine in *Escherichia coli* [78], for which *D. pigrum* already harbors auxotrophies. Additionally, *D. pigrum* seems to be auxotrophic for the vitamins thiamine (vitamin B_1_), riboflavin (vitamin B_2_), and niacin (vitamin B_3_). Vitamin B_1_ has importance for primary carbohydrate and amino acid metabolism [79]. Our analysis further revealed a 4-aminobenzoate, also called p-Aminobenzoate (PABA), auxotrophy. PABA is a component of folate (vitamin B_9_) [80] and, thus, is also associated with the B-vitamins. Rodionov et al. identified transporter proteins for vitamins in various human pathogens, which strictly depend on vitamin uptake [81]. As these transporters are also reported in *D. pigrum*, one could assume that it is also dependent on uptake of the reported B-vitamins. Biochemical experiments are required to confirm all reported auxotrophies.

Having discussed the multiple auxotrophies, it seems apparent that *D. pigrum* has difficulties growing on certain media. The synthetic nasal medium (SNM) and synthetic cystic fibrosis medium (SCFM) mimic two niches, where *D. pigrum* is observed. These habitats, however, are relatively low in nutrient supply. For that reason, metabolites need to be added to the medium definition to enable growth in silico. However, one needs to keep in mind that only single-culture in silico experiments were conducted, combined with in vivo observations. Additional single-culture in vitro growth experiments and coculture in silico experiments might clarify the role of the added metabolites. Sokolovskaya et al. have shown that microbial communities share vitamins. They showed that various mutualisms have evolved between organisms to import and deliver variants of cobamides, including vitamin B_12_ [82]. It needs to be investigated whether the in silico required nutrients are due to the single culture experiments and are obsolete in multi-culture settings. The same was found for the analysis in the nutrient-rich media simulating the blood and gastrointestinal tract.

Comparing the growth rates between the in silico simulations in the SNM and SCFM to the blood and the gastrointestinal medium, one can observe an increased growth rate for the latter two media. This observation seems reasonable, as the blood and the gastrointestinal medium are rich in nutrients that can be taken up and metabolized compared to the media SNM and SCFM.

With our high-quality model, *i*DPM21RW, we were able to confirm predicted auxotrophies and growth behaviors. Laboratory and biochemical experiments as well as additional omics data can be used to further refine this first-time genome-scale metabolic model of *Dolosigranulum pigrum*. This model will pave the way to better understand its metabolism and its interaction and extrusion of the human pathogen *Staphylococcus aureus*.

## 4. Materials and Methods

The first draft reconstruction of *Dolosigranulum pigrum* was initially curated using an automated reconstruction tool. Subsequent automated and manual refinement lead to the first genome-scale metabolic model (GEM) of *D. pigrum*.

### 4.1. Building the Draft Reconstruction

Several tools were used for the draft reconstruction and validation, as explained subsequently.

#### 4.1.1. CarveMe

CarveMe is a fast and automated reconstruction tool for curating genome-scale metabolic models of microbial species and communities [41]. It was used to curate the first draft reconstruction of *D. pigrum* strain 83VPs-KB5. This strain was chosen, as its NCBI assembly level is the only complete genome assembly of *D. pigrum*. Additionally, this strain is the only *D. pigrum* strain in the KEGG database [37]. The coding domain sequence (CDS) of this strain was downloaded from the NCBI assembly database [83], using the accession code ASM19771v1 (RefSeq assembly accession: GCF_007197715.1). With this annotated genome sequence and the default settings of CarveMe version 1.2.2, the initial draft of *D. pigrum* in SBML Level 3 Version 1 format [84] was curated.

#### 4.1.2. ModelPolisher

Subsequently, the ModelPolisher version 2.0.1 was used to annotate the initial draft reconstruction extensively [46]. ModelPolisher matches the identifiers of the model’s entities against the BiGG Models database [45]. For each corresponding entry in BiGG, all available information and data about the matched instance are incorporated as annotations into the initial draft reconstruction. ModelPolisher was run within a docker environment using the additional settings –annotate-with-bigg=true, –add-adb-annotations=true, and –output-combine=true. After this annotation step, all gene–protein reaction (GPR) associations, reaction boundaries, and objective coefficients were unreadable by COBRApy [61] due to inter-conversion difficulties with the SBML flux balance constraints (fbc) package [85]. All unreadable instances were converted to the respective fbc package instances.

#### 4.1.3. Memote

The metabolic model testing suite, Memote determines for each tested GEM an independent and comparable score within a comprehensive overview. Standardized metabolic model tests and the evaluation of a model’s annotations constitute the score. Well-annotated and consistent models have a high Memote score [38]. Each improvement step of the *D. pigrum* model was closely monitored by determining the Memote score in each iteration. Memote was used in its command line version.

### 4.2. Refining the Reconstruction Using Literature Evidence

After the initial draft was curated and annotated, manual refinement steps followed. All manual steps were conducted using COBRApy [61] and libSBML [86].

#### 4.2.1. Mass and Charge Imbalances

The chemical formula and charge were missing for 65 of the metabolites. They were retrieved from the BiGG Models database [45], added to the respective instance, and used to balance reactions in which they participate.

#### 4.2.2. Add Gene Annotations

The ModelPolisher added annotations for all model instances except for the genes. To this point, only the NCBI protein accession numbers from the CDS file were included in the model. A BLAST [62] search was conducted for every NCBI protein accession number to retrieve the respective GenBank [87] identifiers and to increase the gene annotations’ scope. With the help of these GenBank identifiers, the locus tags of the *D. pigrum* genes were identified. These locus tags are also used in the KEGG database [37]. All additionally identified gene annotations were added to the model using libSBML.

#### 4.2.3. Extend Model Manually Using the KEGG Database

Information about *D. pigrum* strain 83VPs KB5 can be found in the KEGG database [37]. The previously retrieved gene annotations were used to compare the already included model genes with the genes listed in the KEGG database to increase the initial reconstruction’s scope. Therefore, the identified metabolic reactions, including GPRs, and probable new metabolites, were added to the model. In the next step, dead-end metabolites were identified. Despite an ortholog and homolog search of related nasal microbes available in the BiGG database, the number of dead-end metabolites could not be decreased. Further genes and reactions were added to the model based on these identified metabolites.

#### 4.2.4. Test for Energy-Generating Cycles

GEMs can contain so-called energy-generating cycles. These cycles are thermodynamically impossible since models with such cycles can charge energy metabolites without nutrient consumption [47]. Fritzemeier et al. suggested a pipeline to identify 14 different energy metabolites, including adenosine triphosphate (ATP), cytidine triphosphate (CTP), guanosine triphosphate (GTP), uridine triphosphate (UTP), inosine triphosphate (ITP), reduced nicotinamide adenine dinucleotide (NADH), NADPH flavine adenine mononucleotide and dinucleotide, ubiquinol-8, menaquinol-8, 2-demethylmenaquinol 8, acetyl-coA, and l-glutamate as well as the proton exchange between cytosol and periplasm. For each metabolite, a dissipation reaction was defined based on Fritzemeier et al. After constraining all uptake reactions to zero, the 15 dissipation reactions were maximized.

#### 4.2.5. Add More Precise SBO Terms

Memote assesses the annotation of model instances with Systems Biology Ontology (SBO) terms [55]. SBO terms provide semantic information about the model instances and allows for explicit and unambiguous understanding of its meaning: the more detailed SBO a term chosen, the more explicit the description given. Metabolites and genes received the general SBO terms for “simple chemical” (SBO:0000247) and “gene” (SBO:0000243), respectively. The reactions’ SBO terms were chosen as precisely as possible using an in-house pipeline [57].

#### 4.2.6. Improve Biomass Objective Function

CarveMe adds a universal biomass equation to the carved model. However, this equation was adapted from the biomass composition of *Escherichia coli* [88] to a universal biomass composition [41,89]. To further improve the biomass objective function (BOF) of the *D. pigrum* reconstruction, BOFdat was used [58]. BOFdat is a Python package to generate and improve a BOF based on organism-specific experimental data. In three steps, the stoichiometric coefficients for (i) the major macromolecules, (ii) inorganic ions and coenzymes, and (iii) the remaining species-specific metabolic biomass precursors are generated and incorporated into the BOF. For refinement of the BOF of *D. pigrum*, its genomic DNA sequence was used as input in the first step. Furthermore, parameters for the dry weight composition are required. Since, at the time of writing, no information about the dry weight composition of *D. pigrum* was available, these parameters were chosen as suggested in the BOFdat documentation. With the DNA sequence and the dry weight composition, the stoichiometric coefficients of the DNA nucleotides deoxyadenosine triphosphate (dATP), deoxythymidine triphosphate (dTTP), deoxyguanosine triphosphate (dGTP), and deoxycytidine triphosphate (dCTP) as well as for diphosphate (ppi) were determined and updated in the BOF. At the time of writing, no transcriptomic, proteomic, or lipidomic data are publicly available. Therefore, the RNA, protein, and lipid macromolecules’ coefficients could not be refined within this work.

After determining the stoichiometric coefficients of the macromolecules, the stoichiometric coefficients of the inorganic ions and coenzymes followed. For this step, the BOFdat script was adapted to run in the latest Python version. All inorganic ions or coenzymes were either added to the BOF, or their stoichiometric coefficients were updated.

Experimental gene essentiality data are required for the inclusion and update of additional species-specific metabolic biomass precursors in step (iii). This step aims to identify condition- and species-specific metabolic end goals. As gene essentiality data are also not publicly available at the time of writing, this step was skipped.

#### 4.2.7. Add ECO Terms

The Evidence and Conclusion Ontology (ECO) comprises classes and terms describing different evidence and assertion methods. These terms capture, e.g., the type of evidence that a gene product or a reaction has. ECO terms are helpful for quality control of a model. For every reaction in the model, the GPR association was extracted. All reactions without a GPR were assigned the ECO term ECO:0000001. This term is defined as an inference from background scientific knowledge. For all remaining genes from the GPRs, the UniProt database [49] was consulted. Protein existences were defined as (i) inferred from homology, (ii) predicted, or (iii) evidence at the transcript level. These existences were assigned to their corresponding ECO terms. All assignments are listed in Table 3. If a GPR consists of only one gene, the corresponding ECO term was added to the reaction. If a reaction had a GPR with multiple genes, the gene with the lowest evidence score was added. The ECO terms in Table 3 are sorted from the lowest to the highest evidence scores. Genes that were not found in the UniProt database were assigned the ECO term ECO:0000251 for the similarity evidence used in the automatic assertion. Hence, if one gene in a GPR with multiple genes was not found in UniProt, the reaction was assigned the lowest evidence score, which is the one for genes not found in UniProt.

All ECO terms were added as annotations with the biological qualifier type BQB_IS_DESCRIBED_BY.

#### 4.2.8. Remove Redundant Information

CarveMe stores information about annotations and other databases in the SBML notes field. However, this information is better stored in the annotations field. Since CarveMe and ModelPolisher use the BiGG Models database, the same annotation information is stored twice: once in the notes by CarveMe and once in the annotations field by the ModelPolisher. To avoid this redundancy and to decrease the file size, the annotation information was removed from the notes field.

#### 4.2.9. Add Subsystems and Groups

With the added annotations, the pathways in which a reaction occurs are included in the model. For every reaction that has an annotated KEGG [37] ID, the KEGG representational state transfer (REST) application programming interface (API) was used to retrieve the associated pathways. These pathways were added as further annotations to the reaction with the biological qualifier type BQB_OCCURS_IN. Furthermore, the “groups” plugin [90], available from SBML Level 3 [59,91], was enabled. Every pathway was defined as a group instance, and every reaction occurring in this pathway was added as a member.

### 4.3. Evaluation and Validation of the Reconstruction

Available knowledge about *D. pigrum* was used and simulated in silico to evaluate and validate *i*DPM21RW as detailed below.

#### 4.3.1. Evaluating Auxotrophies, Biosynthesis Capabilities, and Carbohydrate Metabolism

We mainly used the results from the functional genomic predictions by Brugger et al. [25] to evaluate the auxotrophies and biosynthetic capabilities. All stated auxotrophies were carefully verified by limiting the respective metabolite’s availability and subsequently optimizing the model. If the in silico simulations revealed no growth after limiting the metabolite’s availability, the auxotrophy was considered confirmed. If growth was possible despite the limitation of its availability, the complete biosynthetic pathway of the respective metabolite was evaluated and carefully checked for every individual reaction. Reactions with limited or insufficient genetic proof were removed from the model. For this evaluation step, we mainly relied on literature research, the two databases KEGG [37] and BioCyc [51], and BLAST searches [62]. For predicted reactions and transporters, the model was checked for the presence of the reported reaction and transporters. Missing reactions or transporters were added to the model with its corresponding genes.

#### 4.3.2. Identification of Additional Auxotrophies

A sink reaction for every amino acid was added to identify additional auxotrophies. This sink reaction was maximized after closing the respective exchange reaction to limit its availability. The growth rate was fixed to 0.2 mmol/(gDW · h). As a medium, the self-defined minimal medium was used (see also Section 4.3.4). If no amino acid production or growth was possible after closing the amino acid’s exchange reaction, *D. pigrum* was considered auxotrophic. If the amino acid could be produced, the amino acid production was set in relation to the sole carbon source (d-glucose). The ATP requirement was calculated by summing up all fluxes of ATP-consuming reactions and by putting them in relation to the amino acid production rate. The CO_2_ production rate was computed by setting the CO_2_ transport reaction rate in relation to the amino acid production rate.

#### 4.3.3. Evaluating Growth Capabilities in Different Media

The model *i*DPM21RW was further validated by simulating its growth capabilities in four different environments. The first evaluated habitat was the human nose. For this niche, a chemically defined synthetic nasal medium (SNM) is available [65,72]. As no growth could be simulated with the defined metabolites in the SNM, the identified amino acids for which *D. pigrum* is auxotrophic were added as well. As still no growth was possible, we further evaluated and identified missing components until a growth in the defined medium could be simulated.

This procedure was repeated for the other three media. The synthetic cystic fibrosis medium (SCFM) mimics the lung of patients with CF and was defined by Palmer et al. [66]. For the blood simulations, an adapted medium initially created for the human reconstruction Recon 2.2 [67] was used. The definition for the European diet was extracted from the Virtual Metabolic Human (VMH) database [69,70]. Each metabolite’s exchange reaction and, thus, availability in the analyzed medium was set to 10 mmol/(gDW · h) for determination of the growth rate.

#### 4.3.4. Defining a Minimal Medium for *D. pigrum*

*D. pigrum* holds many requirements for its environment regarding nutrients due to its multiple auxotrophies. We defined a minimal medium specifically for *D. pigrum* to better cultivate this organism in laboratory settings. For this purpose, we used the SNM medium definition and investigated which metabolites could be removed from the medium while maintaining a realistic growth rate. The uptake rate of each metabolites was set to 10 mmol/(gDW · h). The complete list of minimal medium components is given in Table 2.

#### 4.3.5. Evaluating Growth Capabilities on Different Carbon Sources

With the previously defined minimal medium, the in silico growth capabilities of *D. pigrum* on different carbon sources were examined. All available sugar exchange fluxes were extracted from the model and sorted into mono-, di-, and trisaccharides. Each carbon source was tested individually by only enabling the tested carbon source’s exchange reaction and by optimizing the model for growth. Growth was also possible for the available polysaccharides, but these were not further investigated.

### 4.4. Visualization

Escher is a web application for building pathway maps. Reactions, metabolites, and genes can be contextualized within the metabolism of an organism [63]. Besides the web application, an Escher Python package can be run and customized within Jupyter Notebooks [92]. The package can process models using COBRApy [61]. This Python version of Escher was used to draw parts of *D. pigrum*’s metabolism.

## Figures and Tables

**Table 1:** Overview of reported auxotrophies and biosynthetic pathways. Brugger et al. investigated auxotrophies and biosynthetic pathways based on functional genomic predictions [25]. Reported auxotrophies and biosynthesis were verified using *i*DPM21RW and in silico predictions. Additionally, reported reactions and transporters were checked for their presence. A black check-mark (✔) indicates a correct prediction or occurrence of the model’s instance; a check-mark in gray (✔) indicates a correct prediction or occurrence after model modifications; and a black cross (**✗**) indicates a discrepancy between the functional genomic predictions and the model. However, we could not find any discrepancy for auxotrophies and biosynthetic pathways.

	Methionine	Arginine	Glutamine	Putrescine	Spermidine	Biotin	Niacin
Auxotrophy	✔	✔		✔	✔	✔	✔
Biosynthesis			✔				
Reported reactions			✔			✔	✔
Transporter	✔	✔		✔	✔	✔	

**Table 2:** Definition of a minimal medium for *D. pigrum*. Since *D. pigrum* holds many auxotrophies and several requirements for its environment to grow, we defined a minimal medium containing all relevant metabolites. The minimal medium comprises in total 33 compounds, including the 13 amino acids that cannot be produced, 13 trace minerals, d-glucose as a carbon source, and additional vitamins and required compounds.

Amino Acids	Trace Minerals	Other Molecules
l-leucine	Cl^-^ (chloride)	d-glucose
l-threonine	K^+^ (potassium)	4-aminobenzoate
l-arginine	Ca^2+^ (calcium)	riboflavin
l-lysine	Mg^2+^ (magnesium)	thiamine
l-proline	Mn^2+^ (manganese)	niacin
l-glutamate	Co^2+^ (cobalt)	meso-2,6-diaminoheptanedioate
l-histidine	Zn^2+^ (zinc)	O_2_ (oxygen)
l-isoleucine	Cu^2+^ (copper)	
l-methionine	Fe^2+^ (iron II)	
l-tryptophane	Na^+^ (sodium)	
l-valine	Ni^2+^ (nickel)	
l-cysteine	SO_4_^2-^ (sulfate)	
l-phenylalanine	HPO_4_^2-^ (phosphate)	

**Table 3:** ECO terms and their names and assignments. For every Evidence and Conclusion Ontology (ECO) term, the corresponding name is given together with the assignment. ECO terms are ordered in ascending evidence order.

ECO Term	Term Name	Assignment
ECO:0000001	inference from background scientific knowledge	no GPR
ECO:0000251	similarity evidence used in automatic assertion	GPR but no hit in UniProt
ECO:0000363	computational inference used in automatic assertion	UniProt: ‘Predicted’
ECO:0000044	sequence similarity evidence	UniProt: ‘Inferred from homology’
ECO:0000009	transcript expression evidence	UniProt: ‘Evidence at transcript level’

## Data Availability

The genome-scale metabolic model of *D. pigrum* is available in the BioModels Database [93] as an SBML Level 3 Version 1 file [84] within a COMBINE Archive OMEX file [94] at https://www.ebi.ac.uk/biomodels/models, accessed on 7 April 2021 under the accession number MODEL2012220003.

## Supplementary Materials

The following are available at https://www.mdpi.com/article/10.3390/metabo11040232/s1, Figure S1: Metabolic map of *D. pigrum*, Table S2: Stoichiometric coefficients of the biomass objective function (BOF), Table S3: Minimal medium for *D. pigrum*.

## Abbreviations

The following abbreviations are used in this manuscript:

ABC	ATP-binding cassette
API	application programming interface
ATP	adenosine triphosphate
BiGG	Biochemically, Genetically, and Genomically structured
BLAST	Basic Local Alignment Search Tool
BOF	biomass objective function
CDS	coding domain sequence
CF	cystic fibrosis
COBRA	Constraint-Based Reconstruction and Analysis
CTP	cytidine triphosphate
dATP	deoxyadenosine triphosphate
dCTP	deoxycytidine triphosphate
dGTP	deoxyguanosine triphosphate
dTTP	deoxythymidine triphosphate
EC	Enzyme Commission
ECF	energy-coupling factor
ECO	Evidence and Conclusion Ontology
FBA	flux balance analysis
fbc	flux balance constraints
FVA	flux variability analysis
GEM	genome-scale metabolic model
GPR	gene–protein reaction
GTP	guanosine triphosphate
HMDB	Human Metabolome Database
ID	identifier
ITP	inosine triphosphate
KEGG	Kyoto Encyclopedia of Genes and Genomes
MIRIAM	Minimal Information Requested in the Annotation of Models
NADH	reduced nicotinamide adenine dinucleotide
NADPH	reduced nicotinamide adenine dinucleotide phosphat
NCBI	National Center for Biotechnology Information
REST	representational state transfer
SBO	Systems Biology Ontology
SCFM	synthetic cystic fibrosis medium
SNM	synthetic nasal medium
URT	upper respiratory tract
UTP	uridine triphosphate
TCA	tricarboxylic acid
VMH	Virtual Metabolic Human
